# Quantitative Prediction of miRNA-mRNA Interaction Based on Equilibrium Concentrations

**DOI:** 10.1371/journal.pcbi.1001090

**Published:** 2011-02-24

**Authors:** Chikako Ragan, Michael Zuker, Mark A. Ragan

**Affiliations:** 1 ARC Centre of Excellence in Bioinformatics, and Institute for Molecular Bioscience, The University of Queensland, Brisbane, Australia; 2 Rensselaer Polytechnic Institute, Troy, New York, United States of America; Alexander Fleming Biomedical Sciences Research Center, Greece

## Abstract

MicroRNAs (miRNAs) suppress gene expression by forming a duplex with a target messenger RNA (mRNA), blocking translation or initiating cleavage. Computational approaches have proven valuable for predicting which mRNAs can be targeted by a given miRNA, but currently available prediction methods do not address the extent of duplex formation under physiological conditions. Some miRNAs can at low concentrations bind to target mRNAs, whereas others are unlikely to bind within a physiologically relevant concentration range. Here we present a novel approach in which we find potential target sites on mRNA that minimize the calculated free energy of duplex formation, compute the free energy change involved in unfolding these sites, and use these energies to estimate the extent of duplex formation at specified initial concentrations of both species. We compare our predictions to experimentally confirmed miRNA-mRNA interactions (and non-interactions) in *Drosophila melanogaster* and in human. Although our method does not predict whether the targeted mRNA is degraded and/or its translation to protein inhibited, our quantitative estimates generally track experimentally supported results, indicating that this approach can be used to predict whether an interaction occurs at specified concentrations. Our approach offers a more-quantitative understanding of post-translational regulation in different cell types, tissues, and developmental conditions.

## Author Summary

MicroRNAs (miRNAs) are small RNA molecules that regulate post-transcriptional gene expression by binding messenger RNAs (mRNAs), blocking their role in translation or marking them for degradation. To date, computational methods for predicting mRNA targets have assumed an all-or-nothing mode of miRNA-mRNA interaction. Here we introduce a computational approach that predicts the degree of interaction, taking into account initial miRNA and mRNA concentrations. Using this approach, we can predict whether specified interactions are likely to be functionally relevant within physiologically relevant concentration ranges.

## Introduction

miRNAs are short (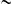
22 nt) endogenous RNAs that exert regulatory control of many cellular processes by suppressing specific mRNAs via complementary base-pairing at a specific target site [1]. It has been suggested that a miRNA can use at least two distinctive mechanisms to regulate protein-coding genes: “switching-off” the entire function of the target gene, and “tuning” the expression level of multiple target genes within appropriate ranges [1]. In the former case, a miRNA reduces the expression of the target mRNA to a level at which the gene can no longer function, potentially leading to observable phenotypes including cell death or abnormal cell phenotypes [2], [3]. In the latter case, a miRNA alters the expression of hundreds of genes to various degrees, maintaining cellular functionality [4].

Each miRNA-mRNA interaction is affected differently by the strength of miRNA-mRNA binding and by the concentration of each interacting species. For example, a specific miRNA might bind to a specific mRNA only if present in high concentration. In tumor cells, some miRNAs are expressed at unusually high or low concentrations [5] and thus may bind more or less extensively to specific mRNAs than in normal cells. The regulation a miRNA exerts on a specific target may also be altered if the concentration of the target mRNA changes during differentiation or development, or as the result of changes in the surrounding environment [6]. Current miRNA prediction methods can predict whether a specific miRNA binds to a specific mRNA, but do not predict whether and how these interactions vary under different concentrations. In this study, we aim not only to predict miRNA-mRNA interactions, but also to estimate their quantitative extent as a function of RNA concentration.

Several distinct algorithmic approaches have been developed to predict miRNA targets. Most require more-or-less stringent base-pair complementarity across a “seed” region (nucleotide positions 2–7 or 2–8 from the 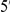 end of miRNA) for miRNA-mRNA duplex formation to be predicted, as implemented in widely used prediction methods such as EMBL [7], miRanda [8], PicTar [9], PITA [10] and TargetScan [11]. Suppression of a target mRNA by a miRNA is mediated by a protein complex referred to as the RNA-induced silencing complex (RISC). A recent study of the crystal structure of this complex shows that the seed region is tightly bound to the complex, emphasizing the importance of seed-matching in recognizing the target site [12].

Other studies show that the efficiency of RNA-RNA (including miRNA-mRNA) interaction is positively correlated with physical accessibility of the target sites [13], [14]. RISC by itself cannot unfold a structured region of mRNA to present a potential target site for interaction with miRNA, although it can promote RNA-RNA annealing [15]. Thus the specificity of miRNA-mRNA interaction involves (at least) two factors: base-pair complementarity between the two interacting RNA species (especially at the seed region), and local folded structure of the potential target mRNA. Target-site accessibility can be assessed in reference to the change in structural energy of the (folded) mRNA before and after a potential target site is opened for interaction with a miRNA. This has led to a two-step hybridization reaction model: first the target site is opened (unfolded) for interaction, then an RNA-RNA duplex is formed at the site [16]. Computational methods to predict mRNAs targeted by miRNAs based on this two-step thermodynamic model have been developed [10], [14].

Here we extend this two-step hybridization reaction model by incorporating another set of factors which critically affect the existence and extent of miRNA-mRNA interactions: concentrations of the interacting molecular species, miRNA and mRNA. On this basis we develop a new method that can estimate the quantitative extent of the interactions. We calculate the equilibrium concentrations of the unbound miRNA, unbound mRNA, and miRNA-mRNA duplex from the initial concentrations of the interacting species and free energies of the interactions.

We apply our method to a set of *Drosophila melanogaster* miRNA-mRNA interactions that have been experimentally tested (including interactions that were successfully confirmed, and those that failed to receive experimental support), and to a set of experimentally supported miRNA-mRNA interactions in human. First, we compare the ability of our method to predict target sites as assessed by sensitivity and specificity, to other methods under the same initial concentrations. Then we test the ability of our method to estimate the degree of interaction (*i.e.* to predict functionally relevant target sites) at the same initial miRNA concentrations used for experimental confirmation. We show that our method can predict target sites at specified concentrations with high accuracy, and that our quantitative estimates generally correlate with experimental results. We also show that some miRNAs can at low concentrations bind to target mRNAs, whereas others are unlikely to bind within a physiologically relevant concentration range.

## Results

### Brief description of our method

Our method consists of three independent components. First we search for potential target sites by predicted free energy of the miRNA-mRNA duplex, rank these results by energy score, and filter this list requiring presence of a seed match. Second, for each identified potential target site, we compute the thermodynamic parameters described in the two-step model [16]. Then we compute the final concentrations of miRNA-mRNA, and the net free energy change (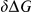) of the interaction based on the initial concentrations of the RNAs.

Instead of the free energy change (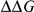) used in the two-step model [16], we use the net free energy change (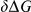) to evaluate the interaction at given initial concentrations (see Materials and Methods). The net free energy change indicates whether a specific interaction occurs. If no interaction occurs between the two species (miRNA and mRNA), the net free energy before and after the interaction does not change, *i.e.* the net free energy change is zero. If an interaction occurs between the miRNA and mRNA, the change will be always negative.

We used FASTH [17], which is computationally scalable for application to transcriptome-scale data, to search for potential target sites and to compute hybridization energies of the miRNA-mRNA duplexes, and UNAFold [18], which adopts the same energy calculation model [19] used in FASTH, to compute mRNA folding energies. Here we introduce Ensemble_Calc to compute the final concentrations of miRNA and mRNA, and the net free energy change (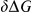) of interaction. The source code of Ensemble_Calc is available at http://mfold.rna.albany.edu/Ensemble.

### Concentrations of miRNAs and mRNAs in a cell

The number of copies of an individual mRNA species present in a single cell is considered to vary over four orders of magnitude (1 to >1000 copies), with most present in <100 copies but a few exceeding 1000 copies [20]. Individual miRNA species are likewise considered to vary widely in copy number per cell, with a few tissue-specific species present more than 10,000 copies per cell [21]. Although miRNA expression varies widely from one miRNA to another, miRNAs are more abundant (average 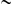
500 copies per cell) than mRNAs [21], and this abundance can help explain the co-regulation of a target mRNA by several miRNAs, and the regulation of multiple mRNAs by a single miRNA. Thus mRNA concentrations in a typical animal cell (1000–25,000 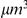 volume) can be as low as about 80 pM (1 copy in a 25,000 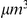 cell) or can exceed 2.2 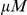 (1000 copies in a 1000 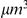 cell), while miRNA concentrations can exceed 22 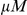 (10,000 copies in a 1000 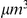 cell) (see Material and Methods).

### Recovery of experimentally tested *D. melanogaster* targets

We applied our model to the set of 190 experimentally tested miRNA-mRNA interactions in *Drosophila melanogaster* reported by Kertesz *et al.* [10]; this set contains both interactions that were successfully confirmed, and those that failed to receieve experimental support. Reporter vectors are usually used to examine whether a miRNA directly represses the expression of a target mRNA by binding to a putative site. Most targets in *Drosophila* have been examined experimentally using reporter vectors, usually with the full-length 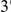
UTR sequence inserted into the vectors [10], [14]; thus their *in vivo* efficiency has been assessed against target structures that are, broadly, similar to those of the native mRNAs. We computed structural energies by folding entire mRNAs where possible; for longer mRNAs it was computationally feasible to fold only the 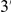
UTR or part of the 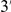
UTR region (see Materials and Methods).

**Figure 1:**
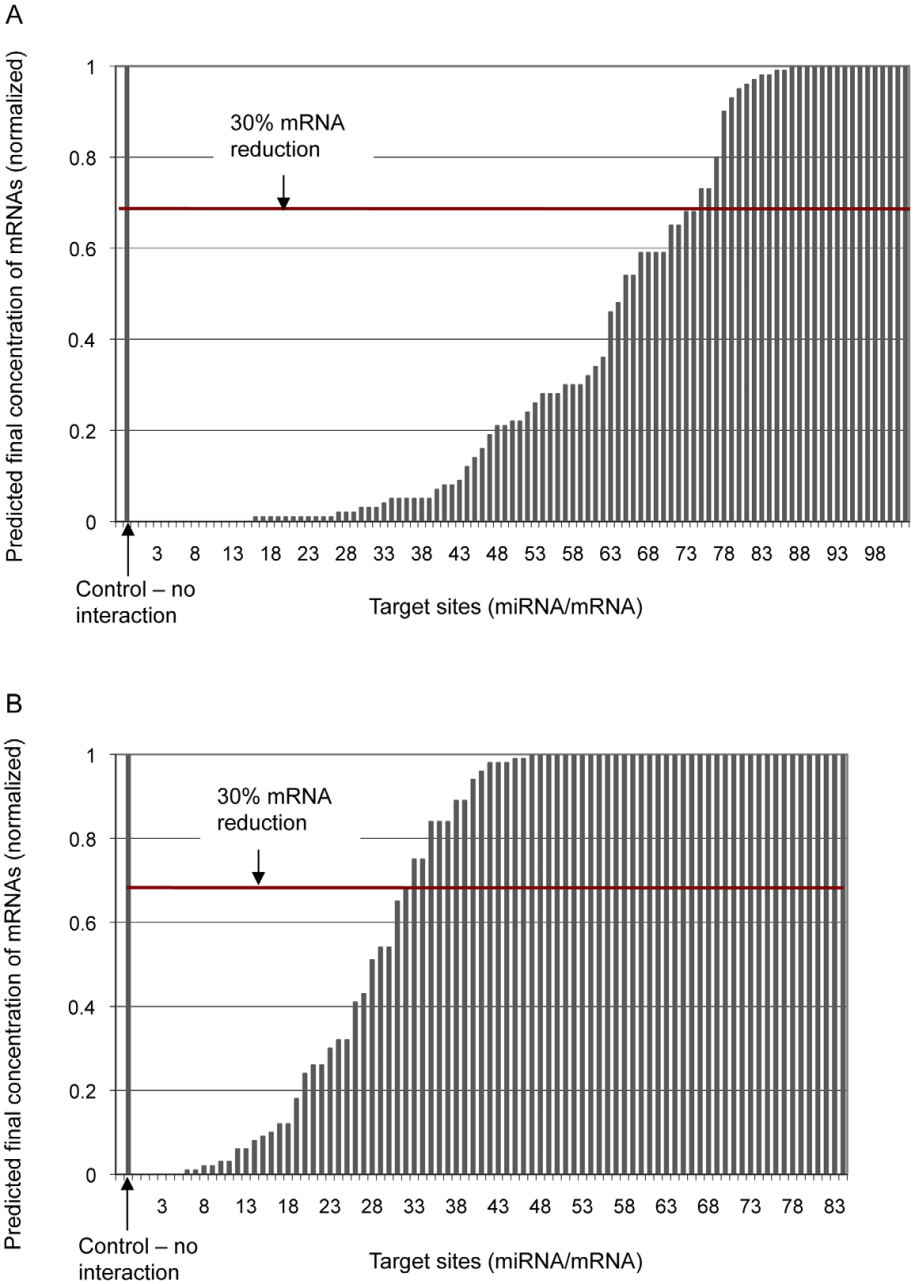
Our method applied to experimentally tested target sites in *Drosophila*. Predicted proportion of mRNA reduction is estimated from the proportion of mRNA remaining unbound for (**a**) 102 confirmed target sites, and (**b**) 84 target sites that failed experimental confirmation at initial concentration of 1
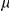
M for both miRNAs and mRNAs. The x-axis shows each miRNA target site, and the y-axis shows the predicted proportion of mRNA remaining unbound after each interaction; *i.e.* if no mRNA has bound to miRNA (no interaction has occurred) the remaining proportion of mRNA is 1 (100%), and if all mRNA has bound to miRNA the remaining proportion is 0 (0%).

Here we assume an initial concentration of 1 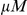 for each miRNA and each mRNA species (see above), and follow common practice in requiring that the predicted mRNA concentration must be reduced by at least 30% for the interaction to be considered functionally relevant (and thus for our prediction to be considered successful). In the following sections we use this criterion as a benchmark to compare with other methods. If we could not identify target sites during the initial search, we assume that no interaction occurs. Using this criterion, our approach recalls 74 (73%) of 102 experimentally confirmed fly miRNAs (Figure 1A and Table S1). Of 88 miRNA-mRNA combinations in fly for which experimental assay failed to confirm an interaction, we were able to obtain the target mRNA sequence from NCBI RefSeq for 84, and for these predict that 52 (62%) do not have a site that is actively bound (Figure 1B and Table S1); based on these data, the sensitivity and specificity of our method are 0.73 and 0.62 respectively.

Since potential sites on an mRNA are usually predicted as either functional (able to be bound by a small RNA, *e.g.* a miRNA) or non-functional (unable to be bound), Kertesz *et al.* [10] applied the standard area under the curve (AUC) to evaluate the sensitivity and specificity of selected existing prediction methods. They observed the highest true-positive rate, 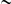
0.79, when the false-positive rate is 0.40; other methods yield true-positive rates of 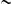
0.64 (MiRanda: [22]), 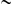
0.71 (PicTar: [23]) and 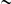
0.74 (EMBL: [24]) at the same 0.40 false-positive rate. We obtained a similar result, observing a 0.73 true-positive rate at 0.38 false-positive rate, using the above criteria.

### Recovery of experimentally confirmed human targets

**Figure 2:**
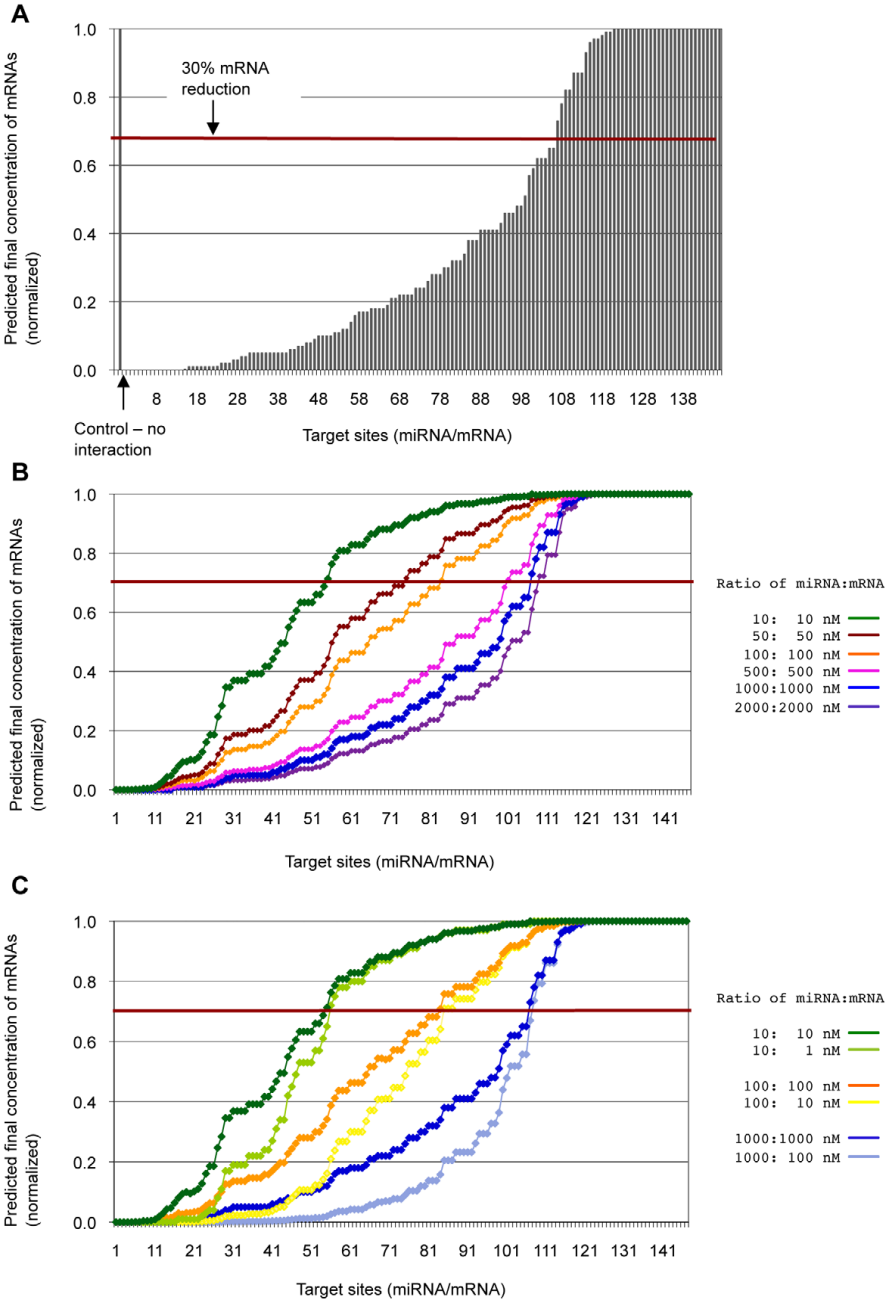
Our method applied to experimentally supported target sites in human. Predicted proportion of mRNA reduction is estimated from the proportion of mRNA remaining unbound for (**a**) 147 experimentally confirmed target sites with initial concentration of 1 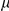
M for both miRNAs and mRNAs, (**b**) with various concentrations (10 nM to 2 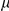
M for both miRNAs and mRNAs), and (**c**) with concentrations of 10 nM, 100 nM and 1 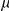
M, with same concentrations for both miRNAs and mRNAs and the ratio of 10∶1, respectively. The x-axis shows each miRNA target site, and the y-axis shows the predicted proportion of mRNA remaining unbound after each interaction; *i.e.* if no mRNA has bound to miRNA (no interaction has occurred) the remaining proportion of mRNA is 1 (100%), and if all mRNA has bound to miRNA the remaining proportion is 0 (0%).

We also investigated whether experimentally supported miRNA binding sites on human mRNAs are predicted using our approach. Unlike the situation in fly (above), human target sites have often been experimentally confirmed by inserting into the reporter vector only the site under investigation, together with short flanking sequences; therefore the energetics of the native mRNA structure has probably not been properly captured in these experiments. Hence, we selected for comparison 147 target sites for which further functional evidence is available. These sites were manually collected (Table S2) based on the following two criteria: the experiment had to be conducted using a reporter gene, and additional validation, such as evidence of inverse correlation between miRNA and target protein expression levels, had to be provided. Of these 147 sites, we predict 106 (72%) to bind to their targets using the criteria described above (Figure 2A and Table S2).

### Total concentration of miRNA affects the degree of interaction

The predicted interactions vary substantially, in extent and properties, among this set of miRNA-mRNA pairs. As shown in Figure 2B, some of these interactions are highly vulnerable to change of concentrations, whereas others are more robust. Furthermore we find that many interactions that can yield a low-energy (strong) duplex are not predicted to do so at typical physiological miRNA concentrations (up to 2 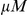).

Until this point we have assumed equal concentrations for both miRNA and mRNA; however, as described earlier, their concentrations are unlikely to be equal. Therefore, we compared the effect of interactions of different initial concentrations of miRNA and mRNA, focusing on situations in which the concentration of miRNA is tenfold greater than that of the mRNA. As shown in Figures 2B and 2C, at 10∶1 we predict slightly greater duplex formation (*i.e.* greater reduction of the level of unbound mRNA) than at equal concentrations (1∶1). The differences occur mostly at the highly efficient target sites, where mRNA concentrations are reduced by more than 50%. Target sites with more-moderate efficiency, where the estimated mRNA reduction is 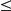
30%, show similar reductions regardless of the ratio of initial concentrations of the two RNA species. Particularly for mRNAs with target sites that saturate quickly, there can be limited scope to reduce their concentration further by increasing the ratio of miRNA to mRNA; increasing the concentration of both species tenfold from 100∶100 to 1000∶1000 (Figure 2B) reduces the mRNA concentration proportionally more than does decreasing the mRNA relative to miRNA from 100∶100 to 100∶10 (Figure 2C). The total concentration of the miRNA has a greater effect on extent of interaction than does the ratio of concentrations.

For some of these experimentally confirmed miRNA-mRNA interactions, we predicted that a single miRNA binds to more than one target site on the 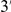
UTRs of a single mRNA, and/or to different transcripts from the gene; in these cases, we use for Figures 1 and 2 the site that yields the greatest reduction in mRNA concentration. Details of predicted target sites with their free energy scores and equilibrium concentrations of unbound miRNA, unbound mRNA and duplex are presented in Tables S1 and S2, and the references are presented in Text S1.

### Quantitative estimates on experimentally confirmed human targets

**Figure 3:**
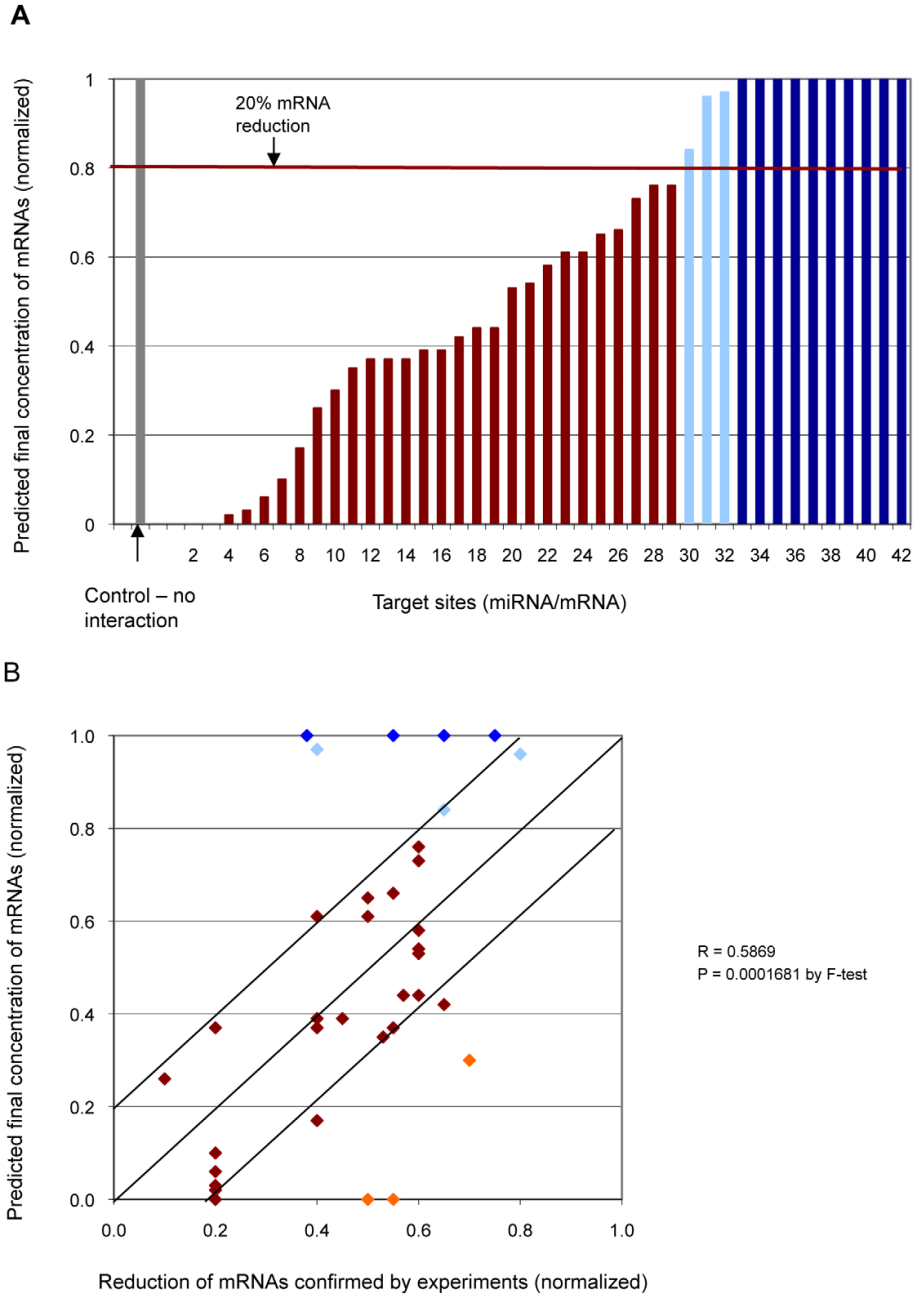
Our method applied to experimentally confirmed target sites in human with specified initial concentration. (**a**) Predicted proportion of mRNA reduction is estimated from the proportion of mRNA remaining unbound for 42 experimentally supported interactions at the same initial concentrations as in the confirmation experiment. The x-axis shows each miRNA target site, and the y-axis shows the predicted proportion of mRNA remaining unbound after each interaction; *i.e.* if no mRNA has bound to miRNA (no interaction has occurred) the remaining proportion of mRNA is 1 (100%), and if all mRNA has bound to miRNA the remaining proportion is 0 (0%). (**b**) Predicted proportion of mRNA reduction compared with 36 experimentally supported interactions at the same initial concentrations as in the confirmation experiment. The x-axis shows the experimentally confirmed and the y-axis show these predicted proportion of mRNA remaining unbound after each interaction. The red and orange dots show successfully predicted target mRNAs, and light blue and dark blue show these unsuccessfully predicted with the initial concentrations specified in the experiment and at 1 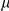
M, respectively.

Among the experimentally supported targets described above in human, miRNA concentrations used for experimental confirmation were reported for 41; one target was confirmed using two different miRNA concentrations (Table S3). These concentrations ranged between 2.5 nM and 300 nM. We tested our model on these 42 interactions, using the reported miRNA concentration and setting the mRNA concentration to be the same. The 41 experimentally supported targets include six sites that we did not recover in our initial search, and four that were recovered but were not predicted to be bound at 1 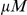 miRNA (above) and are therefore not expected to bind miRNA at lower experimental concentrations. These ten are shown in dark blue in Figure 3A.

If we require that the predicted mRNA concentration be reduced by at least 20% for the interaction to be considered functionally relevant (the same minimum requirement used in the confirmation experiments: [25]), we predict 29 out of 42 interactions (69%) successfully at the miRNA concentration used in each experiment (Figure 3A). In addition to the 10 targets mentioned in the previous paragraph (shown in blue), for three further experimentally supported interactions we predicted that the mRNA concentration is reduced by less than 20% (shown in light blue, Figures 3A and 3B).

For the remaining 35 sites (36 interactions with unique miRNA concentrations) we recovered, the predicted degree of mRNA reduction generally tracks experimental results, with many successfully predicted target sites falling within (or very close to) 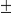
20% of the reported level of reduction (Figure 3B). In cases where we predict that a single miRNA binds to more than one target site on the 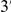
UTR of a single mRNA, and/or to different transcripts from the gene, in Figures 3A and 3B we show only the most energetically favorable interaction. Details of the target sites and associated information are available in Table S3, and the references are available in Text S1.

*In vitro* confirmation experiments are normally carried out in triplicate, and the degree of mRNA reduction of each repeated experiment can vary 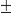
20% from the mean value reported within each study [26]. The degree of mRNA reduction can differ >20% for the same interaction in different type of cells [27]. For the experiments that reported the different mRNA reduction levels in different cell types, we compare our estimates against the mean value of these reported levels (Figure 3B and Table S3).

Since total mRNA concentrations are rarely reported, we set all mRNA concentrations to equal the corresponding miRNA concentrations and examined miRNA-to-mRNA concentration ratios up to 10∶1. As shown in the previous section, in this range our predictions are robust, as assessed by percent recall. As our approach is based on thermodynamic principles, we anticipate its continued applicability under a broader range of physiologically relevant conditions.

Although mRNA destabilization is the major contributor to the repression of target-gene activity [28], [29], some miRNA-mRNA interactions repress translation without destabilizing the mRNAs. Therefore for some interactions, the level of unbound mRNA level may be lower than reported in these experiments. For three sites (shown in orange in Figure 3B), we predicted a much greater degree of mRNA reduction than was reported in the *in vitro* confirmation experiments. Our method estimates the extent to which an mRNA is bound, but cannot predict the outcome of this binding, *i.e.* whether the bound mRNA may or may not be degraded or its translation inhibited.

### Overlap of targets among different prediction methods

As shown above, using the benchmark criteria, the predictive power of our method is similar to those of other methods. We compared the target sites predicted by our method and by miRanda [8], PicTar [9], PITA, PITAtop [10], and TargetScan [11] on 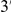
UTRs of human RefSeq mRNAs, using 150 miRNAs for which the targets are predicted by all methods described above. In general, the proportion of overlap among sets of targets predicted by different methods reflects the selection criteria adopted by each method. All of the methods compared here, except PITA, use seed-matching and conservation of target sites across different species as selection criteria, although the definition of seed and the degree of conservation may vary among methods. Therefore the proportion of overlap among their predictions is relatively high (13–77%) (Table S4). miRanda predicts the largest number of targets; 53% of PicTar, 77% of PITAtop and 51% of TargetScan targets are also predicted by miRanda. Maximum overlap is observed between predictions of PITAtop and TargetScan, where over 40% of their predictions overlap. Our method uses seed-matching but not site-conservation as a selection criterion. The overlap between our method and other methods (11–41%) is lower than among other method (13–77%).

PITA, like our method, incorporates site accessibility into its searching mechanism, but the predictions of PITAtop (a list of top predictions produced by the PITA algorithm) include only conserved sites. Overlap between our method and PITAtop is not different from that with other methods compared here. PITA assesses site accessibility; however, the set of PITA targets contains the target sites with positive free-energy changes (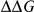) described by the two-step model [16]. We discard those for which the predicted free energy change is 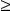
0, then match to the remainder those targets predicted by our method and others. About 91% of our predicted targets are predicted by PITA (overlapped by a PITA-predicted target), slightly higher than for the other methods investigated (85–89%). We summarize these comparisons as shown in Table S4, and the list of our predicted targets is available in Table S5.

We also compared the miRNA targets predicted by five computational methods (including our own) with those identified by Hafner and colleagues [30] using PAR-CLIP. In this approach, cellular mRNAs are cross-linked with the AGO protein complex, and the protein complex is immunoprecipitated; sites of cross-linkage can be revealed by thymidine-to-cytidine transitions in the corresponding cDNAs, and nearby regions of reverse complementarity to miRNA seeds are interpreted as miRNA targets. Applying PAR-CLIP to HEK293 cells, Hafner *et al.* [30] found putative target sites for 98 of the 100 most-abundant miRNAs. Most (72%) of the putative sites identified in this way are imperfectly complementary to miRNA seeds, *i.e.* contain a mismatch or bulge. From these 98 we selected the 68 for which target predictions are available from PicTar, miRanda, PITAtop, TargetScan and from our approach (Table S6), show a perfect WC match at nt 2–7 at the 5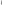 end of miRNAs, and whose clusters can be mapped into 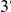
UTRs of RefSeq mRNAs (*i.e.* we compared unique miRNA-mRNA target combinations).

PAR-CLIP predicts many fewer targets than does any of the computational methods discussed here, with overlap ranging from 1.71–1.87% (PicTar, PITAtop and TargetScan) to 1.31% (our method) to 0.87% (miRanda) of the computationally generated predictions. miRanda recovers the largest proportion of PAR-CLIP targets (50%) from 974 predictions; PicTar, PITAtop and TargetScan 29–45% from 566–864 predictions; and our method 20% from 393 predictions. As miRNAs in the PAR-CLIP dataset are highly expressed in HEK293 cells, their concentrations may be greater than our default 1 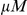. At higher initial miRNA concentrations and the same 30% mRNA reduction threshold we predict 24% (at 2 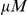) and 27% (4 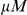) of the PAR-CLIP targets, with this improvement obviously accounted for by lower-affinity sites. Sites with perfect WC matches at nt 2–8 yield similar results as those with WC matches at nt 2–7 (Table S6). Although some of the PAR-CLIP putative target sites that contain WC matches at nt 2–7 may be non-functional, our quantitative results are consistent with the idea that miRNA control is transduced in part through imperfectly complementary sites on mRNAs.

## Discussion

We have developed a computational model that can provide quantitative estimates of RNA-RNA interaction as a function of the concentrations of the interacting species, and have applied our model to predict miRNA-mRNA interactions. Few target sites have been reported with the concentration of total miRNA used in the experiments, necessarily limiting the evaluation of our method. Except as otherwise indicated, we have based our predictions on 1 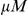 miRNA (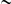
500 copies/cell) [21], and require mRNA levels to be reduced by >30% for the prediction to be considered successful. First we applied our method to experimentally tested *Drosophila* miRNA targets, where positive as well as negative experimental results are available. We predicted these targets with 0.73 sensitivity and 0.62 specificity. By these measures the predictive power of our method is similar to that of these other, widely used methods. Next we applied our method to experimentally confirmed targets in human, and showed that we can achieve similar sensitivity (72%). Then we demonstrated how our method can predict targets at different miRNA concentrations. Using the subset of experimentally suported targets for which total miRNA concentrations are available, we predicted 69% of targets correctly at the specified concentrations and mRNA reduction (requiring >20% reduction). We also showed that our quantitative estimates generally correlated with experimental results; most estimates fall within (or very close to) 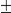
20% of the experimentally corroborated level.

Both known and unknown factors affect miRNA-mRNA interactions and make quantitative estimation difficult. One factor that directly affects the interactions is the cooperative effects of multiple target sites. A target mRNA bound simultaneously by more than one miRNA may show greater repression than one bound at a single site. However, reports suggest that cooperative effects occur only on target sites that are physically proximate, 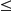
40 nucleotides apart [31], [32]; thus the majority of regulation may be transacted independently through single binding sites. The second factor that directly affects the interaction is the competition among the interactions. Co-expressed miRNAs (and/or other ncRNAs) can likewise compete for mRNA targets; while the binding of each may individually be weak, in a cell in which very many miRNAs are co-expressed there may be a cumulative off-target effect. The different degrees of mRNA reduction observed for the same identified interaction in different cell types, or resulting from transfection of artifical small interfering RNAs (siRNAs) [33] are good examples of these effects. In this study, we present a model of interaction in a simple system that contains two species (one miRNA, one mRNA) that is not able to capture the broad regulations in a systemic way; it will be useful to extend the model to predict the interactions of multiple species of miRNAs and mRNAs simultaneously.

Another factor that may affect the interactions is the self-folded structure of mature miRNA. Since miRNAs are short, the secondary structures of most mature miRNAs are unstable. However a small number of miRNAs can fold into stable structures (hairpins), or can form a homo-dimer duplex with each other [34]. There is also evidence that the secondary structure of (mature) guide siRNA (not precursor structures) also influences the efficiency of siRNA-mRNA interference, where unstructured guide siRNAs confer stronger silencing abilities than structured guide siRNAs [35]. Although our model can incorporate the structure of small RNAs such as miRNA into the free-energy calculation, in this study we did not take secondary structure of each miRNA into account (the structural energy was set to zero), as miRNAs are accommodated into a RISC to interact with target mRNAs. It is nonetheless possible that these stable self- and duplex miRNA structures may affect the incorporation of mature miRNAs into a RISC, perhaps making some of them unavailable for interaction with mRNAs.

Our results indicate that absolute concentration of miRNAs can be important for regulation. It has been reported that the concentration of a miRNA must exceed a threshold in order for a target mRNA to be suppressed [36]. The two species must furthermore be expressed at the same spatiotemporal location at the same time. Expression profiles of all RNA species should be described in absolute concentrations [6], as (for example), the same relative tenfold change from 1 nM to 10 nM may have significantly different biological outcomes than from 100 nM to 1 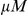.

Some interactions are robust and can regulate the target mRNAs at low concentrations; other interactions are predicted to be concentration-sensitive within the expected range of physiologically relevant concentrations, while yet others are predicted not to occur at all within physiologically likely concentrations. Computational approaches have proven valuable for predicting which mRNAs can be targeted by a given miRNA; however, although other methods predict which mRNAs can be targeted, they do not capture the sensitivity of the predicted interaction to concentrations of reactants. Incorporating concentration into thermodynamically based miRNA target prediction thus can provide finer-grained prediction while avoiding the artificiality of *a priori* thresholds.

## Materials and Methods

### Data

miRNA sequences were obtained from miRBase release 14.0 [37] (www.sanger.ac.uk/software/Rfam), and NCBI RefSeq mRNA sequences (mrnaRefseq.txt) were obtained from UCSC (hgdownload.cse.ucsc.edu/downloads.html). mRNAs were mapped to gene annotations using the refFlat files also from UCSC, and the rna.gbff files downloaded from NCBI (ftp.ncbi.nih.gov/refseq).

### Thermodynamic model

The interaction between a short RNA (*e.g.* miRNA or siRNA) and an entire mRNA has been modelled as a competition between all folded states of the mRNA with or without hybridization of the short RNA to a particular location of the mRNA. The free energy of the folded states of the mRNA in the absence of hybridization is denoted by 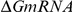. If the short RNA binds to a particular location, then 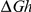 denotes the hybridization free energy of the short RNA binding to its target in the mRNA. Since the target site cannot interact with other bases of the mRNA, an additional computation yields 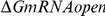, the free energy of the restricted folded states of the mRNA where the target site is single-stranded, or open. The change in free energy (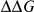) when the short RNA hybridizes to the mRNA is given by
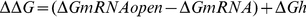
(1)


The target sites for the above computations are chosen in two stages. In the first stage, we ignore folding of the mRNA, and consider only those target sites for which the hybridization free energy (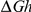) is “sufficiently negative”; *i.e.* for which the miRNA forms an energetically favorable duplex with the target mRNA, where “favorable” is assessed against a subjectively chosen energy threshold. The second computation finds target regions that are accessible for hybridization (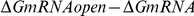). In this way, suitable target sites are chosen by the change of the free energy [16].

The distributions of finite-length DNA or RNA molecules in a solution can be described as an ensemble of all possible polynucleotide sequences pairs of mixed species, such as single-folded strands and double-stranded hybridizations [38]. Here we assume that interactions between two or more mRNAs, and hybridization of short RNAs to each other, do not occur, since there is no reported evidence that such interactions directly afffect interactions between miRNA and mRNA. Then the distribution of a short RNA (miRNA) and an mRNA in a contained system can be described as a combination of the folded state of the mRNA, the hybridization of the short RNA to the mRNA (if any), and the un-folded state of the short RNA.

If S (Short) and T (Target) denote the short RNA (miRNA) and the target mRNA, respectively, then [S] and [T] denote their equilibrium (final) concentrations respectively, and [ST] the equilibrium concentration of the hybridized molecular species. Also, [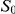] and [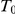] denote the total (initial) strand concentrations of S and T respectively. Conservation of mass yields
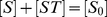
(2)and
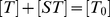
(3)At equilibrium,
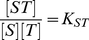
(4)where 
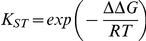
, *R* is the gas constant (1.987 *K*cal/mol) and T is the temperature in  *K*.

Solving equations (2), (3) and (4) for [S] and [T], a numerically stable formula for [S] is given by
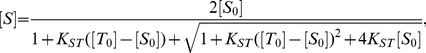
(5)similarly,
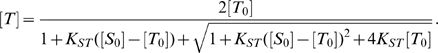
(6)When [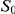] = [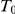] the solutions simplify to
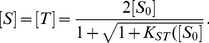
(7)Let 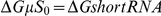 be the potential energy of a short RNA in its initial state, and 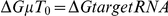 be the potential energy of a target RNA in its initial state. Then the total (ensemble) energy of the system in the initial state (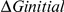) is
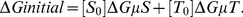
(8)


We also can compute the potential energy of a short RNA in its equilibrium state (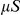), and the potential energy of a target RNA in its equilibrium state (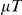), as
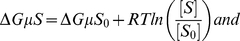
(9)

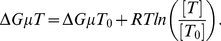
(10)


Then the total (ensemble) free energy of the system at equilibrium state (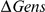) is
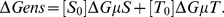
(11)


The net free energy change (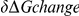) of the interaction is obtained as
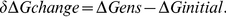
(12)


If no interaction occurs, the net free energy change is zero, and if an interaction occurs the net free energy change is always negative, as the interaction is a spontaneous process.

### Prediction of target sites and computation of equilibrium concentrations

As described previously, our method consists of three independent components.

#### Initial target site prediction by duplex free energies

We used the FASTH program [17] to identify the initial potential target sites that exhibit optimal and near-optimal free energies in 3
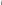
UTRs for both fly and human. Then we filtered the potential target sites by setting further criteria such as requiring Watson-Crick matches at seed regions (nt 2–7) for the targets in human, or allowing one GU pair in the seed region for the targets in fly. Energetically unfavorable canonical pairs (*e.g.* tandem A∶U G∶U pairs) may be counted as mismatches by FASTH. We allowed one GU pair in the seed region, as is commonly done for fly [10], [22], [23] and sometimes [8], [10] but not always [9], [10], [11] for human and other mammals.

For any energetically suboptimal duplex to be considered, we requirie it to have a free energy (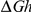) of maximum −8.5 kcal/mol. If the energy is >−8.5 kcal/mol, the site becomes a target (achieving mRNA reduction >30% at the concentration <1 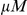) only if the accessibility is very high (the difference between 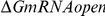 and 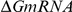 must be close to zero). Only the target sites that meet the above criteria were processed further.

#### Computation of local folding energies of the target mRNAs

We used the hybrid-ss-min program (free energy minimization) from UNAFold [18] to compute the structural energy of the (folded) target mRNAs (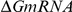), and the structural energy was constrained by forcing the mRNA to be unpaired at the predicted target sites (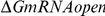). For mRNAs 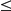
6000 nt in length, we computed structural energies by folding the entire mRNA, except for a small number of mRNAs that took a very long time to process, for which we folded only the 3
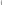
UTR. For all mRNAs >6000 nt, we folded only the 3
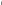
UTR. The very few 3
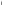
UTRs of >6000 nt were divided into slightly overlapping sections (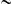
4000 nt per section) that we folded separately.

The preliminary determination of likely targets, and the more-intensive computations of mRNA folding energies described above, may be accomplished by using different approaches, including partition functions or free energy minimization. Partition functions yield likely target sites through the computation of stochastic samples. Free energy methods do the equivalent by selecting hybridizations that can occur within any prescribed free energy increment from the minimum. While it has been said that the predictions of secondary structures of large RNAs such as mRNAs are poor [39], we do not need to estimate an entire mRNA structure correctly. What we have to compute is the free energy difference between no hybridization, and hybridization to a particular target site. For this purpose, using energy minimization and partition functions yield similar results; also, folding a short sequences (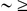
800 nt) flanking the target site and folding an entire mRNA yield similar predictions [40]. However, we recommend that different methods (and energy rules) not be mixed for these calculations, *i.e.* if an energy-minimization method with a nearest-neighbor energy model is used to compute the hybridization energy between the two RNA species, then the same method should be used to compute the folding energies; in particular, the fixed-energy model and the nearest-neighbor energy model shold not be mixed. Different models produce different hybridization or folding energies for the same site. Since we compute energy change from multiple free energy values, each free energy has to be obtained using a consistent underlying rule, as otherwise the value of the free energy change is unreliable.

Although here we have used FASTH [17] and UNAFold [18], which minimize energies based on the nearest-neighbor energy model [19], other software can be used as well as to obtain the free energy parameters needed to compute equilibrium concentrations.

#### Computation of equilibrium concentrations and net free energy change

The software Ensemble_calc computes the equilibrium (final) concentrations of miRNA, mRNA and miRNA-mRNA in molar concentrations, and the net free energy change (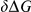) of the interaction. Application of this program requires parameter values to be set for the free energies 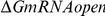, 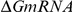 and 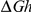 as described above, and initial concentrations (in moles) of the miRNA and mRNA to be specified by the user.

The FASTH source code is available by request from MZ (zukerm@rpi.edu). Both the UNAFold and Ensemble_Calc can be downloaded from http://mfold.rna.albany.edu/.

### Estimation of concentration in a single cell

We estimated the molar concentration of miRNA and mRNA species from the number of RNA copies expressed in a single cell as follows. Given that typical animal cells are 10–30 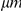 on an edge, assuming a cubical shape and assuming that the nucleus occupies 25% of the volume, the molar concentration (C) in a cell (in a cytoplasm) can be calculated as follows:
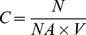
(13)where N is the number of copies of an RNA species, V is the volume of cytoplasm in a cell, and NA is Avogadro's number. For example, the molar concentration of an RNA species present at 1 copy in a cell of 30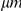 edge is
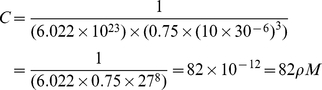
(14)Similarly, the molar concentration of an RNA species present at 1000 copies in cell of 10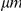 edge is
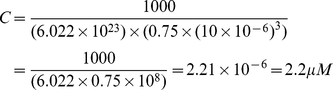
(15)Considering that the cytoplasm in actual cells is replete with organelles, membranes and other structures that occupy volume, the actual concentrations of RNA species may be several-fold higher than the above numbers suggest.

## Supporting Information

Table S1 List of our predictions on experimentally tested targets in fly (*Drosophila melanogaster*) with initial concentrations of 1 µM for both miRNA and mRNA. The selected target sites shown in Figure 1 are indicated in red. The miRNA, mRNA, and miRNA-RNA duplex concentrations are normalized equilibrium (final) concentrations.
(0.08 MB XLS)

Table S2 List of our predictions on experimentally supported targets in human with initial concentrations of 1 µM for both miRNA and mRNA. The selected targets sites shown in Figure 2 are indicated in red. The miRNA, mRNA, and miRNA-mRNA duplex concentrations are normalized equilibrium (final) concentrations.
(0.08 MB XLS)

Table S3 List of our predictions on experimentally supported targets in human with specified initial concentrations. The predictions are made with the same initial miRNA concentration used in each experiment. The targets that are not predicted by our method using the same concentration but predicted with 1 µM concentration are indicated in light blue, and the sites that are not predicted with 1 µM concentrations are indicated in blue. The miRNA, mRNA, and miRNA-mRNA duplex are normalized equilibrium (final) concentrations.
(0.03 MB XLS)

Table S4 Comparison with other target prediction methods. Our predictions are constructed using the target sites that achieved a >30% mRNA reduction with the initial concentrations of 1 µM for both miRNA and mRNA. Each target consists of a unique (non- redundant) interaction (miRNA-mRNA).
(0.04 MB DOC)

Table S5 List of target sites used for the comparison with other methods. The list contains multiple target sites, if any, for each interaction (miRNA-mRNA).
(5.55 MB TXT)

Table S6 Degree of overlap between PAR-CLIP prediction sets and those of other methods including ours.
(0.05 MB DOC)

Text S1 Description of material for supplemental tables, including additional references.
(0.05 MB DOC)
