# Supplementary material for: Quantitative Prediction of miRNA-mRNA Interaction Based on Equilibrium Concentrations
Source: PLoS Comput Biol. 2011 Feb 24;7(2):e1001090. doi: 10.1371/journal.pcbi.1001090 (PMC3044769; doi:10.1371/journal.pcbi.1001090)
Supplement: Table S4 — Comparison with other target prediction methods. Our predictions are constructed using the target sites that achieved a >30% mRNA reduction with the initial concentrations of 1 µM for both miRNA and mRNA. Each target consists of a unique (non- redundant) interaction (miRNA-mRNA). (0.04 MB DOC) [file pcbi.1001090.s004.doc]

**Supplemental Table S4**: Degree of overlap between our prediction sets and those of other methods

The Table shows the number of miRNA targets in human 3′ UTRs predicted by our method and PicTar [1], MiRanda [2], PITA and PITAtop [3] and TargetScan [4], and the number and percent that are also predicted by each other method.

|  |  | **PicTar** | | **miRanda** | | **PITAtop** | | **TargetScan** | | **Our method** | |
| --- | --- | --- | --- | --- | --- | --- | --- | --- | --- | --- | --- |
|  |  | **Num** | **%** | **Num** | **%** | **Num** | **%** | **Num** | **%** | **Num** | **%** |
|  | Num targets | 55940 |  | 232116 |  | 89732 |  | 77168 |  | 60284 |  |
| PicTar | 55940 |  |  | 29652 | 53 | 28196 | 50 | 21874 | 39 | 10245 | 18 |
| **miRanda** | 232116 | 29652 | 13 |  |  | 68971 | 30 | 39543 | 17 | 24762 | 11 |
| **PITAtop** | 89732 | 28196 | 31 | 68971 | 77 |  |  | 36633 | 41 | 15840 | 18 |
| **PITA** | 1098794 | 47459 | 85 | 203354 | 88 |  |  | 68750 | 89 | 54855 | 91 |
| **TargetScan** | 77168 | 21874 | 28 | 39543 | 51 | 36633 | 47 |  |  | 14059 | 18 |
| **Our method** | 60284 | 10245 | 17 | 24762 | 41 | 15840 | 26 | 14059 | 23 |  |  |

**References:**

1. Krek A, Grun D, Poy MN, Wolf R, Rosenberg L *et al.* (2005) Combinatorial microRNA target predictions. *Nat Genet* 37:495-500.

The data set was downloaded from the online Supplemental Material.

2. John B, Enright AJ, Avavin A, Tuschl T, Sander C *et al.* (2004) Human microRNAs targets. *PLoS Biol* 2:e363.

miRanda version 3.0 was downloaded from http://www.microrna.org

3.Kertesz M, Iovino N, Unnerstall U, Gaul U, Segal E (2007) The role of site accessibility in microRNA target recognition. *Nat Genet* 39:1278-1284.

PITA and PITAtop version 6 were downloaded from http://genie.weizmann.ac.il/pubs/mir07/mir07_data.html

4. Friedman RC, Farh KK, Burge CB, Bartel DP (2009) Most mammalian mRNAs are conserved Targets of microRNAs. *Genome Research* 19:92-105.

TargetScan version 5.1 was downloaded from http://www.targetscan.org/
