# Supplementary material for: Quantitative Prediction of miRNA-mRNA Interaction Based on Equilibrium Concentrations
Source: PLoS Comput Biol. 2011 Feb 24;7(2):e1001090. doi: 10.1371/journal.pcbi.1001090 (PMC3044769; doi:10.1371/journal.pcbi.1001090)
Supplement: Table S6 — Degree of overlap between PAR-CLIP prediction sets and those of other methods including ours. (0.05 MB DOC) [file pcbi.1001090.s006.doc]

**Supplemental Table S6**: Degree of overlap between PAR-CLIP prediction sets and those of other methods including ours

The 68 human miRNAs, for which targets are described in this Table, are those for which targets (3′UTRs of human mRNAs) were predicted by all the methods considered: PAR-CLIP [1], PicTar [2], MiRanda [3], PITAtop [4], TargetScan [5] and our method (this paper). From left to right, the columns in Table S6 show: column 1, method name (and for our method, concentration of miRNA and mRNA); column 2, numbers of targets predicted by each method; column 3, the number of predicted targets within the set of 1927 targets predicted by PAR-CLIP when Watson-Crick pairing is required at nucleotide positions 2-7 inclusive; columns 4 and 5, the corresponding percentages of the 1927 PAR-CLIP predictions and of the number of targets predicted by that method (from column 2); and columns 6-8, as for columns 3-5 only based on the 1247 targets predicted by PAR-CLIP requiring WC pairing at positions 2-8 inclusive.

For the 100 most-abundant miRNAs in HEK293 cells, PAR-CLIP predicted 38221 cross-linked sites [1] which can show different modes of binding with the miRNA seed regions, *i.e.* some contain a mismatch or a bulge. Of these 38221 sites, we selected the 10860 that contain WC seed matches corresponding to nt 2-7 of the miRNA, and mapped them to 3′UTRs of RefSeq mRNAs, yielding 2467 sites of which 2326 represent non-redundant miRNA-mRNA combinations. Of these 2326 non-redundant PAR-CLIP targets, 1927 are predicted as targets of the 68 miRNAs described in the paragraph above. Similarly, 1247 targets are predicted for these 68 miRNAs when a WC seed match is required at miRNA positions 2-8.

| Column 1 | Column 2 | Column 3 | Column 4 | Column 5 | Column 6 | Column 7 | Column 8 |
| --- | --- | --- | --- | --- | --- | --- | --- |
| Method | Num targets | PAR-CLIP  nt 2-7 | % PAR-CLIP | % Num | PAR-CLIP  nt 2-8 | % PAR-CLIP | % Num |
| PicTar | 31638 | 566 | 29.37 | 1.79 | 457 | 36.65 | 1.44 |
| miRanda | 112009 | 974 | 50.54 | 0.87 | 816 | 65.44 | 0.73 |
| PITAtop | 48992 | 840 | 43.59 | 1.71 | 757 | 60.71 | 1.55 |
| TargetScan | 46274 | 864 | 44.84 | 1.87 | 709 | 56.86 | 1.53 |
| Our method |  |  |  |  |  |  |  |
| 1 µM 30%* | 30008 | 393 | 20.39 | 1.31 | 297 | 23.82 | 0.99 |
| 2 µM 30%* | 32852 | 454 | 23.56 | 1.38 | 336 | 26.94 | 1.02 |
| 4 µM 30%* | 35589 | 519 | 26.93 | 1.46 | 389 | 31.19 | 1.09 |

* Targets predicted at initial concentrations 1 µM (2 µM, 4 µM) for each miRNA and each mRNA species, and requiring the predicted mRNA concentration to be reduced by at least 30%.

1. Hafner M, Landthaler M, Burger L, Khorshid M, Hausser J *et al.* (2010) Transcriptome-wide identification of RNA-binding protein and microRNA target sites by PAR-CLIP. *Cell* 141:129-141.

The data set was downloaded from the supplemental materials.

2. Krek A, Grun D, Poy MN, Wolf R, Rosenberg L *et al.* (2005) Combinatorial microRNA target predictions. *Nat Genet* 37:495-500.

The dataset was downloaded from the online Supplemental Materials.

3. John B, Enright, AJ, Avavin A, Tuschl T, Sander C *et al.* (2004) Human microRNAs targets. *PLoS Biol* 2:e363.

miRanda version 3.0 was downloaded from http://www.microrna.org

4.Kertesz M, Iovino N, Unnerstall, U, Gaul, U, Segal E. (2007) The role of site accessibility in microRNA target recognition. *Nat Genet* 39:1278-1284.

PITA and PITAtop version 6 were downloaded from http://genie.weizmann.ac.il/pubs/mir07/mir07_data.html

5. Friedman RC, Farh KK, Burge CB, Bartel DP (2009) Most mammalian mRNAs are conserved targets of microRNAs. *Genome Research* 19:92-105.

TargetScan version 5.1 was downloaded from http://www.targetscan.org/
