# Supplementary material for: Quantitative Prediction of miRNA-mRNA Interaction Based on Equilibrium Concentrations
Source: PLoS Comput Biol. 2011 Feb 24;7(2):e1001090. doi: 10.1371/journal.pcbi.1001090 (PMC3044769; doi:10.1371/journal.pcbi.1001090)
Supplement: Text S1 — Description of material for supplemental tables, including additional references. (0.05 MB DOC) [file pcbi.1001090.s007.doc]

# Material for Supplemental Tables

**Table S1**

List of our predictions on experimentally tested targets in fly (*Drosophila melanogaster*) with initial concentrations of 1 μM for both miRNA and mRNA. The selected target sites shown in Figure 1 are indicated in red. miRNA, mRNA, and miRNA/mRNA concentrations are normalized equilibrium (final) concentrations.

## Table S2

List of our predictions on experimentally supported targets in human with initial concentrations of 1 μM for both miRNA and mRNA. The selected targets sites shown in Figure 2 are indicated in red. miRNA, mRNA, and miRNA/mRNA concentrations are normalized equilibrium (final) concentrations.

## Table S3

List of our predictions on experimentally supported targets in human with specified initial concentrations. The predictions are made with the same initial miRNA concentration (concentration) used in each experiment. The targets that are not predicted by our method using the same concentrations but predicted with 1 μM concentrations are indicated in light blue, and the sites that are not predicted with 1 μM concentrations are indicated in blue. miRNA, mRNA, and miRNA/mRNA concentrations are normalized equilibrium (final) concentrations.

## Table S4

Comparison with other target prediction methods. Our predictions are constructed using the targets sites that achieved a > 30% mRNA reduction with the initial concentrations of 1 μM for both miRNA and mRNA. Each target consists a unique (non redundant) interaction (miRNA-mRNA).

## Table S5

A list of target sites that is used for the comparison with other methods. The list contains multiple target sites, if any, for each interaction (miRNA-mRNA).

**Table S6**

Degree of overlap between PAR-CLIP prediction sets and those of other methods including ours.

# References to Table S1

Brennecke J, Hipfner DR, Stark A, Russell RB, Cohen SM (2003) *bantam* encodes a developmentally regulated microRNA that controls cell proliferation and regulates the proapoptotic gene *hid* in *Drosophila*. Cell : 25-36.

Brennecke J, Stark A, Russell RB, Cohen SM (2005) Principles of microRNA-target recognition. PLoS Biol. 3: e85.

Burgler C, Macdonald PM (2005) Prediction and verification of microRNA targets by MovingTargets, a highly adaptable prediction method. BMC Genomics 6: 88.

Kertesz M, Iovino N, Unnerstall U, Gaul U, Segal E (2007) The role of site accessibility in microRNA target recognition. Nat. Genet. 39: 1278-1284.

Kwon C, Han Z, Olson EN, Srivastava D (2005) MicroRNA1 influences cardiac differentiation in Drosophila and regulates Notch signaling. Proc. Natl Acad. Sc.i USA 102: 18986-91.

Lai EC, Tam B, Rubin GM (2005) Pervasive regulation of Drosophila Notch target genes by GY-box-, Brd-box-, and K-box-class microRNAs. Genes Dev. 19: 1067-1080.

Leaman D, Chen PY, Fak J, Yalcin A, Pearce M. et al. (2005) Antisensemediated depletion reveals essential and specific functions of microRNAs in Drosophila development. Cell 121: 1097-108.

Li X, Carthew RW (2005) A microRNA mediates EGF receptor signaling and promotes photoreceptor differentiation in the Drosophila eye. Cell 123: 1267-1277.

Li Y, Wang F, Lee JA, Gao FB. (2006) MicroRNA-9a ensures the precise specification of sensory organ precursors in Drosophila. Genes Dev. 20: 2793-2805.

Rehwinkel J, Natalin P, Stark A, Brennecke J, Cohen SM. et al. (2006) Genome-wide analysis of mRNAs regulated by Drosha and Argonaute proteins in *Drosophila melanogaster*. Mol. Cell. Biol. 26: 2965-2975.

Robins H, Li Y, Padgett RW (2005) Incorporating structure to predict microRNA targets. Proc. Natl Acad. Sci. USA 102: 4006-4009.

Ronshaugen M, Biemar F, Piel J, Levine M, Lai EC (2005) The *Drosophila* microRNA iab-4 causes a dominant homeotic transformation of halteres to wings. Genes Dev 19: 2947-2952.

Stark A, Brennecke J, Russell RB, Cohen SM (2003) Identification of *Drosophila* MicroRNA targets. PLoS Biol. 1: E60.

Stark A, Brennecke J, Bushati N, Russell RB, Cohen SM (2005) Animal microRNAs confer robustness to gene expression and have a significant impact on 3'UTR evolution. Cell 123: 1133-1146.

Teleman AA, Maitra S, Cohen SM (2006) *Drosophila* lacking microRNA miR-278 are defective in energy homeostasis. Genes Dev 20: 417-422.

# References to Supplemental S2 and S3

Anderson C, Catoe H, Werner R (2006) MIR-206 regulates connexin43 expression during skeletal muscle development. Nucl. Acids Res. 34: 5863-5871.

Bhattacharya R, Nicoloso M, Arvizo R, Wang E, Cortez A, et al. (2009) MiR-15a and miR-16 control Bmi-1 expression in ovarian cancer. Cancer Res 69: 9090-95.

Bommer GT, Gerin I, Feng Y, Kaczorowski AJ, Kuick R, et al. (2007) p53-Mediated Activation of miRNA34 Candidate Tumor-Suppressor Genes. Curr Biol 17: 1298-307.

Boutz PL, Chawla G, Stoilov P, Black DL (2007) MicroRNAs regulate the expression of the alternative splicing factor nPTB during muscle development. Genes Dev 21: 71-84.

Bueno MJ, Pérez de Castro I, Gómez de Cedrón M, Santos J, Calin GA, et al. (2008) Genetic and epigenetic silencing of microRNA-203 enhances ABL1 and BCR-ABL1 oncogene expression. Cancer Cell 13: 496-506.

Carraro G, El-Hashash A, Guidolin D, Tiozzo C, Turcatel G, et al. (2009) miR-17 family of microRNAs controls FGF10-mediated embryonic lung epithelial branching morphogenesis through MAPK14 and STAT3 regulation of E-Cadherin distribution. Dev Biol. 333: 238-250.

Castellano L, Giamas G, Jacob J, Coombes RC, Lucchesi W, et al. (2009) The estrogen receptor-alpha-induced microRNA signature regulates itself and its transcriptional response. Proc Natl Acad Sci USA 106: 15732-15737.

Chan SY, Zhang YY, Hemann C, Mahoney CE, Zweier JL, et al. (2009) MicroRNA-210 controls mitochondrial metabolism during hypoxia by repressing the iron-sulfur cluster assembly proteins ISCU1/2. Cell Metab. 10: 273-84.

Chen JF, Mandel EM, Thomson JM, Wu Q, Callis TE, et al. (2006) The role of microRNA-1 and microRNA-133 in skeletal muscle proliferation and differentiation. Nat Genet 38: 228-233.

Chen XM, Splinter PL, O'Hara SP, LaRusso NF. (2007) A cellular micro-RNA, let-7i, regulates Toll-like receptor 4 expression and contributes to cholangiocyte immune responses against Cryptosporidium parvum infection. J Biol Chem. 282: 28929-28938.

Cimmino A, Calin GA, Fabbri M, Iorio MV, Ferracin M, et al. (2005) miR-15 and miR-16 induce apoptosis by targeting BCL2. Proc Natl Acad Sci USA 102: 13944-133949.

Cordes KR, Sheehy NT, White MP, Berry EC, Morton SU, et al. (2009) miR-145 and miR-143 regulate smooth muscle cell fate and plasticity. Nature 460: 705-10.

Crawford M, Batte K, Yu L, Wu X, Nuovo GJ, et al. (2009) MicroRNA 133B targets pro-survival molecules MCL-1 and BCL2L2 in lung cancer. Biochem Biophys Res Commun. 388: 483-439.

Davis BN, Hilyard AC, Lagna G, Hata A (2008) SMAD proteins control DROSHA-mediated microRNA maturation. Nature 454: 56-61.

Du C, Liu C, Kang J, Zhao G, Ye Z, et al. (2009) MicroRNA miR-326 regulates TH-17 differentiation and is associated with the pathogenesis of multiple sclerosis.

Nat Immunol. 10:1252-9.

Fazi F, Rosa A, Fatica A, Gelmetti V, De Marchis ML et al. (2005) A minicircuitry comprised of microRNA-223 and transcription factors NFI-A and C/EBPalpha regulates human granulopoiesis. Cell 123: 819-831.

Felli N, Fontana L, Pelosi E, Botta R, Bonci D et al. (2005) MicroRNAs 221 and 222 inhibit normal erythropoiesis and erythroleukemic cell growth via kit receptor down-modulation. Proc Natl Acad Sci USA 102: 18081-18086.

Fontana L, Pelosi E, Greco P, Racanicchi S, Testa U, et al.(2007) MicroRNAs 17-5p-20a-106a control monocytopoiesis through AML1 targeting and M-CSF receptor upregulation. Nat Cell Biol. 9:775-87.

Fujita S, Ito T, Mizutani T, Minoguchi S, Yamamichi N, et al.(2008) miR-21 Gene expression triggered by AP-1 is sustained through a double-negative feedback mechanism.

J Mol Biol. 378: 492-504.

Garzon R, Pichiorri F, Palumbo T, Iuliano R, Cimmino A et al. (2006) MicroRNA fingerprints during human megakaryocytopoiesis. Proc Natl Acad Sci USA 103: 5078-5083.

Gramantieri L, Ferracin M, Fornari F, Veronese A, Sabbioni S, et al. (2007) Cyclin G1 is a target of miR-122a, a microRNA frequently down-regulated in human hepatocellular carcinoma. Cancer Res. 67: 6092-6099.

Gramantieri L, Fornari F, Ferracin M, Veronese A, Sabbioni S, et al. (2009) MicroRNA-221 targets Bmf in hepatocellular carcinoma and correlates with tumor multifocality. Clin Cancer Res. 15: 5073-5081.

Guo LM, Pu Y, Han Z, Liu T, Li YX, et al. (2009) MicroRNA-9 inhibits ovarian cancer cell growth through regulation of NF-kappaB1. FEBS J. 276: 5537-5546.

He L, He X, Lim LP, de Stanchina E, Xuan Z, et al. (2007) A microRNA component of the p53 tumour suppressor network. Nature 447: 1130-1134.

He X, Duan C, Chen J, Ou-Yang X, Zhang Z, et al. (2009) Let-7a elevates p21(WAF1) levels by targeting of NIRF and suppresses the growth of A549 lung cancer cells. FEBS Lett. 583: 3501-3507.

Horie T, Ono K, Nishi H, Iwanaga Y, Nagao K, et al. (2009) MicroRNA-133 regulates the expression of GLUT4 by targeting KLF15 and is involved in metabolic control in cardiac myocytes. Biochem Biophys Res Commun. 389: 315-320.

Hossain A, Kuo MT, Saunders GF (2006) Mir-17-5p regulates breast cancer cell proliferation by inhibiting translation of AIB1 mRNA. Mol Cell Biol. 26: 8191-8201.

Hou J, Wang P, Lin L, Liu X, Ma F, et al. (2009) MicroRNA-146a feedback inhibits RIG-I-dependent Type I IFN production in macrophages by targeting TRAF6, IRAK1, and IRAK2. J Immunol. 183: 2150-2158.

Huang YW, Liu JC, Deatherage DE, Luo J, Mutch DG, et al. (2009) Epigenetic repression of microRNA-129-2 leads to overexpression of SOX4 oncogene in endometrial cancer. Cancer Res. 69: 9038-9046.

Iliopoulos D, Hirsch HA, Struhl K (2009) An epigenetic switch involving NF-kappaB, Lin28, Let-7 MicroRNA, and IL6 links inflammation to cell transformation. Cell 139: 693-706.

Iorio MV, Casalini P, Piovan C, Di Leva G, Merlo A, et al. microRNA-205 regulates HER3 in human breast cancer. Cancer Res 69: 2195-2200.

Itoh T, Nozawa Y, Akao Y (2009) MicroRNA-141 and -200a are involved in bone morphogenetic protein-2-induced mouse pre-osteoblast differentiation by targeting distal-less homeobox 5. J Biol Chem. 284: 19272-19279.

Johnson SM, Grosshans H, Shingara J, Byrom M, Jarvis R, et al. (2005) RAS is regulated by the let-7 microRNA family. Cell 120: 635-674.

Kato M, Zhang J, Wang M, Lanting L, Yuan H, et al. (2007) MicroRNA-192 in diabetic kidney glomeruli and its function in TGF-beta-induced collagen expression via inhibition of E-box repressors. Proc. Natl Acad. Sci. U S A. 104: 3432-3437.

Kong W, Yang H, He L, Zhao JJ, Coppola D, et al. (2008) MicroRNA-155 is regulated by the transforming growth factor beta/Smad pathway and contributes to epithelial cell plasticity by targeting RhoA. Mol Cell Biol. 28: 6773-6784.

Labbaye C, Spinello I, Quaranta MT, Pelosi E, Pasquini L, et al. (2008) A three-step pathway comprising PLZF/miR-146a/CXCR4 controls megakaryopoiesis. Nat Cell Biol. 10: 788-801.

Lei Z, Li B, Yang Z, Fang H, Zhang GM, et al. (2009) Regulation of HIF-1alpha and VEGF by miR-20b tunes tumor cells to adapt to the alteration of oxygen concentration. PLoS One 4: e7629.

Li Y, Guessous F, Zhang Y, Dipierro C, Kefas B, et al. (2009) MicroRNA-34a inhibits glioblastoma growth by targeting multiple oncogenes. Cancer Res. 69: 7569-7576.

Lukiw WJ, Zhao Y, Cui JG (2008) An NF-kappaB-sensitive micro RNA-146a-mediated inflammatory circuit in Alzheimer disease and in stressed human brain cells. J Biol Chem. 283: 31315-31322.

Makeyev EV, Zhang J, Carrasco MA, Maniatis T (2007) The MicroRNA miR-124 promotes neuronal differentiation by triggering brain-specific alternative pre-mRNA splicing. Mol Cell. 27: 435-448.

Manni I, Artuso S, Careccia S, Rizzo MG, Baserga R, et al. (2009) The microRNA miR-92 increases proliferation of myeloid cells and by targeting p63 modulates the abundance of its isoforms. FASEB J. 23: 3957-3966.

Martin MM, Lee EJ, Buckenberger JA, Schmittgen TD, Elton TS (2006) MicroRNA-155 regulates human angiotensin II type 1 receptor expression in fibroblasts. J. Biol. Chem. 281: 18277-18284.

Mayr C, Hemann MT, Bartel DP (2007) Disrupting the pairing between let-7 and Hmga2 enhances oncogenic transformation. Science 315: 1576-1579.

Meng F, Henson R, Lang M, Wehbe H, Maheshwari S, et al. (2006) Involvement of human micro-RNA in growth and response to chemotherapy in human cholangiocarcinoma cell lines. Gastroenterology 130: 2113-2129.

Naguibneva I, Ameyar-Zazoua M, Polesskaya A, Ait-Si-Ali S, Groisman R, et al. (2006) The microRNAs miR-181 targets the homobox protein Hox-A11 during mammalian myoblast differentiation. Nat. Cell Biol. 8: 278-284.

Neilson JR, Zheng GX, Burge CB, Sharp PA (2007) Dynamic regulation of miRNA expression in ordered stages of cellular development. Genes Dev. 21: 578-589.

Noonan EJ, Place RF, Pookot D, Basak S, Whitson JM, et al. (2009) miR-449a targets HDAC-1 and induces growth arrest in prostate cancer. Oncogene. 28: 1714-1724.

O’Donnell KA, Wentzel EA, Zeller KI, Dang CV, Mendell JT (2005) c-Myc-regulated microRNAs modulate E2F1 expression. Nature 435: 839-843.

Ohgawara T, Kubota S, Kawaki H, Kondo S, Eguchi T, et al. (2009) Regulation of chondrocytic phenotype by micro RNA 18a: involvement of Ccn2/Ctgf as a major target gene. FEBS Lett. 583: 1006-1010.

Pan YZ, Morris ME, Yu AM. (2009) MicroRNA-328 negatively regulates the expression of breast cancer resistance protein (BCRP/ABCG2) in human cancer cells. Mol Pharmacol. 75: 1374-1379.

Papagiannakopoulos T, Shapiro A, Kosik KS (2008) MicroRNA-21 targets a network of key tumor-suppressive pathways in glioblastoma cells. Cancer Res. 68: 8164-8172.

Pedersen IM, Otero D, Kao E, Miletic AV, Hother C, et al. (2009) Onco-miR-155 targets SHIP1 to promote TNFalpha-dependent growth of B cell lymphomas. EMBO Mol Med.1: 288-295.

Poy MN, Eliasson L, Kurtzfeldt J, Kuwajima S, Ma X, *et al.* (2004) A pancreatic islet-specific microRNAs regulates insulin secretion. Nature 432:226-230.

Reddy SD, Ohshiro K, Rayala SK, Kumar R (2008) MicroRNA-7, a homeobox D10 target, inhibits p21-activated kinase 1 and regulates its functions. Cancer Res. 68:8195-200.

Reddy SD, Pakala SB, Ohshiro K, Rayala SK, Kumar R. (2009) MicroRNA-661, a c/EBPalpha target, inhibits metastatic tumor antigen 1 and regulates its functions. Cancer Res. 69: 5639-5642.

Rodriguez A, Vigorito E, Clare S, Warren MV, Couttet P, et al. (2007) Requirement of bic/microRNA-155 for normal immune function. Science 316: 608-611.

Romania P, Lulli V, Pelosi E, Biffoni M, Peschle C, et al. (2008) MicroRNA 155 modulates megakaryopoiesis at progenitor and precursor level by targeting Ets-1 and Meis1 transcription factors. Br J Haematol. 143: 570-580.

Rosenberg MI, Georges SA, Asawachaicharn A, Analau E, Tapscott SJ (2006) MyoD inhibits Fstl1 and Utrn expression by inducing transcription of miR-206. J. Cell Biol.175: 77-85.

Saito Y, Liang G, Egger G, Friedman JM, Chuang JC, et al. (2006) Specific activation of microRNA-127 with downregulation of the proto-oncogene BCL6 by chromatin-modifying drugs in human cancer cells. Cancer Cell 9: 435-443.

Salvi A, Sabelli C, Moncini S, Venturin M, Arici B, et al. (2009) MicroRNA-23b mediates urokinase and c-met downmodulation and a decreased migration of human hepatocellular carcinoma cells. FEBS J. 276: 2966-2982.

Sander S, Bullinger L, Klapproth K, Fiedler K, Kestler HA, et al. (2008) MYC stimulates EZH2 expression by repression of its negative regulator miR-26a. Blood 112: 4202-4012.

Saydam O, Shen Y, Würdinger T, Senol O, Boke E, et al. (2009) Downregulated microRNA-200a in meningiomas promotes tumor growth by reducing E-Cadherin and activating the Wnt/ß-catenin signaling pathway. Mol. and Cellular Bio. 29: 5923-5940.

Sætrom P, Biesinger J, Li SM, Smith D, Thomas LF, et al. (2009) A risk variant in an miR-125b binding site in BMPR1B is associated with breast cancer pathogenesis. Cancer Res. 69: 7459-7465.

Scott GK, Goga A, Bhaumik D, Berger CE, Sullivan CS, et al. (2006) Coordinate suppression of ERBB2 and ERBB3 by enforced expression of micro-RNA miR-125a or miR-125b. J. Biol. Chem. 282: 1479-1486.

Segura MF, Hanniford D, Menendez S, Reavie L, Zou X, et al. (2009) Aberrant miR-182 expression promotes melanoma metastasis by repressing FOXO3 and microphthalmia-associated transcription factor. Proc Natl Acad Sci USA 106: 1814-1819.

Song G, Zhang Y, Wang L. (2009) MicroRNA-206 targets notch3, activates apoptosis, and inhibits tumor cell migration and focus formation. J Biol Chem. 284: 31921-31927.

Strum JC, Johnson JH, Ward J, Xie H, Feild J, et al. (2009) MicroRNA 132 regulates nutritional stress-induced chemokine production through repression of SirT1. Mol Endocrinol. 23: 1876-1884.

Tan Z, Randall G, Fan J, Camoretti-Mercado B, Brockman-Schneider R, et al. (2007) Allele-specific targeting of microRNAs to HLA-G and risk of asthma. Am J Hum Genet. 81: 829-34.

Tardif G, Hum D, Pelletier JP, Duval N, Martel-Pelletier J (2009) Regulation of the IGFBP-5 and MMP-13 genes by the microRNAs miR-140 and miR-27a in human osteoarthritic chondrocytes. BMC Musculoskelet Disord.10: 148.

Tavazoie SF, Alarcón C, Oskarsson T, Padua D, Wang Q, et al. (2008) Endogenous human microRNAs that suppress breast cancer metastasis. Nature. 451:147-52.

Tsuchiya Y, Nakajima M, Takagi S, Taniya T, Yokoi T (2006) MicroRNA regulates the expression of human cytochrome P450 1B1. Cancer Res. 66: 9090-9098.

Tuddenham L, Wheeler G, Ntounia-Fousara S, Waters J, Hajihosseini MK, et al. (2006) The cartilage specific microRNA-140 targets histone deacetylase 4 in mouse cells. FEBS Lett. 580: 4214-4217.

Vo N, Klein ME, Varlamova O, Keller DM, Yamamoto T, et al. (2005) A cAMP-response element binding protein-induced microRNA regulates neuronal morphogenesis. Proc Natl Acad Sci USA 102:16426-16431.

Volinia S, Calin GA, Liu CG, Ambs S, Cimmino A, et al. (2006) A microRNA expression signature of human solid tumors defines cancer gene targets. Proc Natl Acad Sci USA 103: 2257-2261.

Voorhoeve PM, le Sage C, Schrier M, Gillis AJ, Stoop H, et al. (2006) A genetic screen implicates miRNA-372 and miRNA-373 as oncogenes in testicular germ cell tumors. Cell 124: 1169-1181.

Wang F, Fu XD, Zhou Y, Zhang Y (2009) Down-regulation of the cyclin E1 oncogene expression by microRNA-16-1 induces cell cycle arrest in human cancer cells. BMB Rep. 42: 725-730.

Wang P, Zou F, Zhang X, Li H, Dulak A, et al. (2009) microRNA-21 negatively regulates Cdc25A and cell cycle progression in colon cancer cells. Cancer Res. 69: 8157-8165.

Welch C, Chen Y, Stallings RL (2007) MicroRNA-34a functions as a potential tumor suppressor by inducing apoptosis in neuroblastoma cells. Oncogene 26: 5017-5022.

Xiao J, Yang B, Lin H, Lu Y, Luo X, et al. (2007) Novel approaches for gene-specific interference via manipulating actions of microRNAs: examination on the pacemaker channel genes HCN2 and HCN4. J Cell Physiol 212: 285-292.

Xu N, Papagiannakopoulos T, Pan G, Thomson JA, Kosik KS (2009) MicroRNA-145 regulates OCT4, SOX2, and KLF4 and represses pluripotency in human embryonic stem cells. Cell 2009 137: 647-58.

Yamakuchi M, Ferlito M, Lowenstein CJ (2008) miR-34a repression of SIRT1 regulates apoptosis. Proc Natl Acad Sci USA 105: 13421-13426.

Yan D, Dong Xda E, Chen X, Wang L, Lu C, et al. (2009) MicroRNA-1/206 targets c-Met and inhibits rhabdomyosarcoma development. J Biol Chem 284: 29596-29604.

Yang X, Feng M, Jiang X, Wu Z, Li Z, et al. (2009) miR-449a and miR-449b are direct transcriptional targets of E2F1 and negatively regulate pRb-E2F1 activity through a feedback loop by targeting CDK6 and CDC25A. Genes Dev. 23: 2388-2393.

Yekta S, Shih, I, Bartel DP (2004) MicroRNA-directed cleavage of HOXB8 mRNA. Science 304: 594-596.

Zaidi SK, Dowdy CR, van Wijnen AJ, Lian JB, Raza A, et al. (2009) Altered Runx1 Subnuclear Targeting Enhances Myeloid Cell Proliferation and Blocks Differentiation by Activating a miR-24/MKP-7/MAPK Network. Cancer Res 69: 8249-8255.

Zhang Y, Chao T, Li R, Liu W, Chen Y, et al. (2009) MicroRNA-128 inhibits glioma cells proliferation by targeting transcription factor E2F3a. J Mol Med 87: 43-51.

Zhao Y, Samal E, Srivastava D (2005) Serum response factor regulates a muscle-specific microRNAs that targets Hand2 during cardiogenesis. Nature 436: 214-220.

Zhao Y, Ransom JF, Li A, Vedantham V, von Drehle M, et al. (2007) Dysregulation of cardiogenesis, cardiac conduction, and cell cycle in mice lacking miRNA-1-2. Cell 129: 303-317.

Zhao JJ, Lin J, Yang H, Kong W, He L, et al. (2008) MicroRNA-221/222 negatively regulates estrogen receptor alpha and is associated with tamoxifen resistance in breast cancer. J Biol Chem 283: 31079-1086.

Zhu S, Si ML, Wu H, Mo YY (2007) MicroRNA-21 targets the tumor suppressor gene tropomyosin 1 (TPM1). J Biol Chem 282: 14328-14336.
